# Changes in Patient-Reported Symptoms Across Chemotherapy Cycles: A Prospective Cohort Study in an Outpatient Chemotherapy Clinic

**DOI:** 10.3390/nursrep16090307

**Published:** 2026-08-28

**Authors:** Maria Lavdaniti, Ioanna Tsatsou, Anastasia Asimakopoulou, Antzouletta Kampitsi, Antonia Vasilopanagi, Mpara Ourania, Polixeni Liamopoulou, Theocharis I. Konstantinidis, George Tzavelas

**Affiliations:** 1 Nursing Department, International Hellenic University, 57400 Thessaloniki, Greece; tzeni_liam@ihu.gr; 2 Nursing Department, University of West Attica, 12243 Athens, Greece; itsatsou@uniwa.gr; 3 Department of Statistics and Insurance Science, University of Piraeus, 18534 Piraeus, Greece; anastasia502@hotmail.com (A.A.); tzafor@unipi.gr (G.T.); 4 Atlas Medical, 15344 Athens, Greece; akampitsi@atlasmedical.gr; 5 Anticancer Hospital “Agios Savvas”, 15351 Athens, Greeceraniampara@yahoo.gr (M.O.); 6 Nursing Department, Hellenic Mediterranean University, 71410 Heraklion, Greece

**Keywords:** chemotherapy, symptom assessment, ESAS-R, oncology nursing, symptom burden, patient-reported outcomes

## Abstract

**Background/Objectives:** Cancer patients undergoing treatment experience a wide range of physical and psychological symptoms that significantly impact their quality of life. This study aimed to evaluate the occurrence and intensity of symptoms in cancer patients using the Edmonton Symptom Assessment System Revised (ESAS-R) during two distinct phases of treatment. **Methods**: A prospective cohort study with consecutive recruitment of eligible patients within a convenience sample was conducted in a tertiary public cancer hospital in Athens, Greece. The initial sample included 189 adult cancer patients assessed after the third chemotherapy cycle. Of these, 105 patients completed a second assessment after the final chemotherapy cycle and constituted the longitudinal follow-up sample. Symptom intensity was measured using the 10-item ESAS-R self-report tool. **Results**: The study population was predominantly female (77.2%), married (71.4%), and diagnosed with breast cancer (59.3%). Statistically significant changes between the two measurements (*p* < 0.05) were observed in pain, fatigue, and well-being. Total symptom burden scores remained relatively stable, slightly decreasing from 25.09 to 24.19. Age showed a consistent negative correlation with pain (*p* < 0.001) in the second measurement and nausea across both measurements (*p* = 0.004 and *p* = 0.017), indicating that younger patients reported higher symptom intensity. Logistic regression indicated that employment/housework was primarily linked to the total symptom score in Measurement A (*p* = 0.010), with patients less likely to have high symptom severity (Exp(B) = 0.101). Baseline symptom burden category at Measurement A was identified as a significant predictor of symptom intensity at Measurement B (*p* = 0.028), with patients presenting mild symptom burden at baseline being less likely to report moderate-to-high symptom burden at the final assessment. **Conclusions**: While pain, fatigue, and perceived well-being deteriorated from the early to the final chemotherapy cycle, the total symptom burden remained relatively stable. Younger patients appeared to experience higher symptom burden, while lower educational level was associated with higher symptom burden in the univariate analysis at the final assessment, highlighting the need for age-sensitive and educationally tailored nursing interventions to enhance symptom management in oncology settings.

## 1. Introduction

Cancer is a leading global health challenge and a major cause of morbidity and mortality worldwide. It is recognized as the second most significant contributor to premature mortality globally [1]. Cancer patient loads in hospitals are increasing day by day [2] and new innovative treatments arise, enhancing survival [3]. Nevertheless, conventional treatments such as chemotherapy remain a cornerstone of modern oncology, widely used across cancer types and stages [4]. However, its treatment is consistently associated with a substantial symptom burden for patients [5].

Symptom burden is a specific concept and describes the total impact of the symptoms experienced by patients, encompassing their presence, frequency, severity, and the level of distress they cause, on daily functioning and quality of life [6]. Also, rather than isolated side effects, individuals undergoing chemotherapy often experience symptom clusters where multiple symptoms occur simultaneously and interact with one another [7]. Cancer symptoms extend beyond the physical domain, such as pain, fatigue, nausea, and sleep disturbances [8], to include significant psychological challenges, including anxiety, depression, and emotional distress [9]. Together, they create a multidimensional burden that can profoundly compromise patients’ quality of life and daily functioning. This complexity creates a cumulative distress that requires systematic, holistic assessment to guide effective nursing interventions [10].

Although previous studies in the Greek oncology setting have provided valuable information on symptom burden, much of the available evidence has been based on cross-sectional assessments, limiting the evaluation of symptom trajectories across chemotherapy treatment phases. Cross-sectional designs provide only a snapshot of patients’ experiences and do not capture the clinical trajectory of symptom burden across treatment phases. This longitudinal gap limits the ability of oncology nurses to provide proactive, stage-specific supportive care. Therefore, this study aimed to evaluate the occurrence and intensity of physical and psychological symptoms using the ESAS-R across early and late chemotherapy phases and to identify evolving predictors of total symptom burden. We hypothesized that age and treatment-related factors would be associated with symptom burden and that the relative influence of demographic and clinical factors might differ across treatment phases.

## 2. Materials and Methods

### 2.1. Design, Participants, and Setting

A prospective cohort study was conducted in a tertiary public cancer hospital in Athens, Greece. The study included adult cancer patients undergoing chemotherapy in an outpatient/day-care chemotherapy setting. Eligible patients were consecutively approached by members of the research team during their scheduled visits to the outpatient/day-care chemotherapy unit. Thus, although recruitment was consecutive, the resulting sample was a non-probability convenience sample. Recruitment was conducted from April 2021 to April 2022.

**Figure 1:**
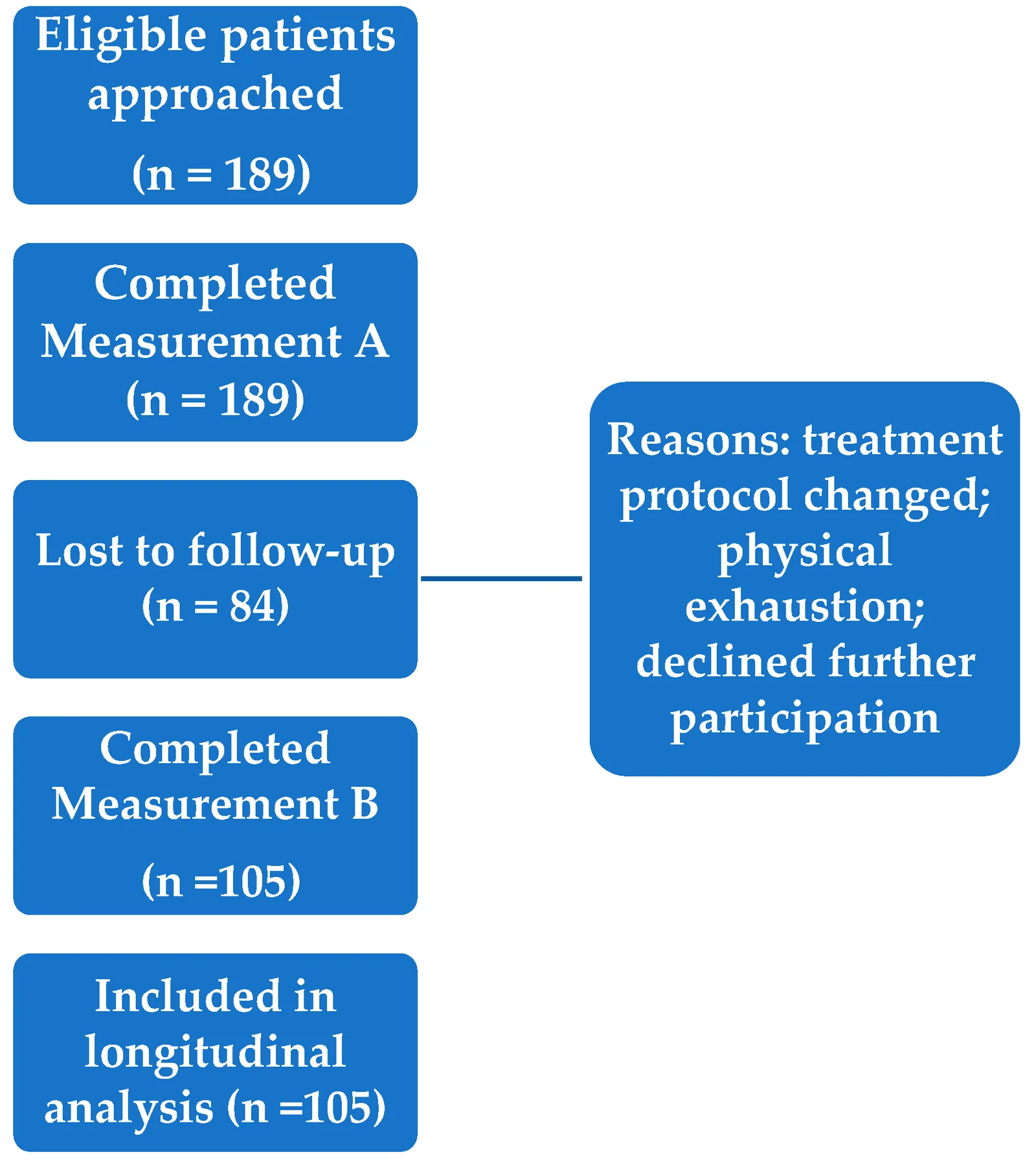
Participant selection diagram.

Patients were assessed at two points: Measurement A, after the third chemotherapy cycle, and Measurement B, after the final chemotherapy cycle (Figure 1). The interval between Measurement A (after the third chemotherapy cycle) and Measurement B (after the final chemotherapy cycle) depended on the individual chemotherapy protocol. The initial sample consisted of 189 patients at Measurement A. Of these, 105 patients completed both assessments and constituted the longitudinal follow-up sample included in the paired analysis at Measurement B. Therefore, the patients assessed at Measurement B represent a subset of the initial Measurement A cohort. Of the 189 participants enrolled at baseline, 105 completed the follow-up assessment.

Eligible participants were adult cancer patients receiving anticancer treatment, including chemotherapy alone or in combination with immunotherapy or targeted therapies. Patients were excluded from the study if they had significant cognitive impairment or psychiatric disorders that hindered their ability to accurately self-assess their symptoms. Additionally, individuals were excluded if they were unable to read, write, or understand the Greek language sufficiently to complete the self-report questionnaires independently. Patients who were physically unable to complete the forms, even with minor assistance, or those who declined to provide informed consent were also excluded from the final analysis.

Eligible patients were consecutively approached by members of the research team while attending the outpatient day-care chemotherapy unit for their scheduled chemotherapy appointments. Recruitment took place during the study period after patients had completed at least three chemotherapy cycles. Potential participants were screened for eligibility according to the predefined inclusion and exclusion criteria. Patients who met the eligibility criteria received verbal and written information about the study and were given the opportunity to ask questions before providing written informed consent. Following consent, participants completed the study questionnaires in the chemotherapy unit before or during treatment administration. Patients who agreed to participate at Measurement A were invited to complete the same questionnaire again after their final chemotherapy cycle.

No formal a priori sample size calculation was performed because this was a prospective cohort study using a convenience sample of all eligible patients attending the outpatient chemotherapy clinic during the study period. The final sample size was determined by the number of eligible patients who consented to participate.

Nevertheless, with 105 participants completing both assessments, a two-sided significance level of 0.05, and 80% statistical power, the sensitivity analysis indicated that the achieved sample could detect a standardized within-participant effect size of approximately *d_z_* = 0.28. This value represents the minimum detectable effect under the specified assumptions and should not be interpreted as an a priori validation of sample-size adequacy. The longitudinal sample was therefore considered adequate for detecting changes of this magnitude, although smaller differences may have remained undetected. The larger baseline sample of 189 participants was used for descriptive and cross-sectional analyses.

The attrition rate between Measurement A and Measurement B was 44.4%. To assess whether attrition introduced systematic bias, a non-responder analysis was performed by comparing the baseline characteristics of patients who completed both measurements (*n* = 105) with those who did not complete the follow-up assessment (*n* = 84). No statistically significant differences were found between the two groups in terms of age, gender, or baseline total symptom burden score (*p* > 0.05), suggesting that the follow-up sample remained broadly comparable to the initial cohort. The main reasons for attrition included treatment protocol changes and physical exhaustion that prevented further participation.

### 2.2. Data Collection

At each measurement point, patients completed a demographic and clinical data form, as well as the Edmonton Symptom Assessment System Revised (ESAS-R).

The questionnaires were self-administered in the outpatient chemotherapy unit before or during treatment administration. Participants completed the ESAS-R independently; when necessary, minor assistance was provided only to clarify the instructions or recording procedure and did not involve suggesting or influencing responses.

The ESAS-R is a validated, 10-item self-report tool used in palliative care and chronic illness management to assess the severity of common physical and psychological symptoms, including pain, fatigue, nausea, depression, anxiety, and well-being. Utilizing a 0–10 numerical rating scale; where 0 represents absence and 10 the worst possible severity, the tool allows patients to rate symptoms “now” to provide a consistent, longitudinal profile of symptom burden. Higher scores indicate greater symptom intensity and distress. By tracking changes over time, the ESAS-R enables healthcare providers to monitor symptom stability, guide clinical interventions, and enhance quality of life, especially in hospice or tertiary settings. The scale provides a total symptom burden score by summing the numerical ratings of the nine core symptoms (pain, tiredness, nausea, depression, anxiety, drowsiness, appetite, shortness of breath, and well-being), with potential scores ranging from 0 to 90, where higher scores indicate a greater total symptom burden [11,12,13].

### 2.3. Data Analysis

Statistical analysis was performed using the software package SPSS 26.0 (IBM Corp., released 2019, IBM SPSS Statistics for Windows, v.26.0, Armonk, NY, USA: IBM Corp.). All statistical tests were two-tailed, and a *p*-value of less than 0.05 was considered statistically significant.

A per-protocol analysis was adopted for the longitudinal comparison, focusing on patients who completed both Measurement A and Measurement B. A complete-case analysis was performed, whereby participants with missing data for variables included in a specific analysis were excluded from that analysis. No imputation of missing values was undertaken. In addition, a non-responder analysis was conducted to assess whether attrition introduced systematic bias between the two measurement points.

Descriptive analyses were conducted using all available observations at each measurement point. For each ESAS-R item, the mean and standard deviation were calculated using the valid responses for that item. The Total Symptom Burden Score was calculated at the participant level by summing the scores of the nine core ESAS-R items, excluding the “other problem” item. The mean and standard deviation of these participant-level total scores were then calculated separately for Measurement A and Measurement B. No missing values were imputed. Because the number of valid observations could vary across individual ESAS-R items and the participant-level total score, the denominators used for these calculations could differ.

Longitudinal paired comparisons were conducted only among the 105 patients who completed both measurements. The Wilcoxon signed-rank test was used to compare symptom scores between Measurement A and Measurement B. The Mann–Whitney U test was performed to compare symptom values between two independent groups, such as gender or previous chemotherapy experience, while the Kruskal–Wallis H test was used for variables with more than two categories, including marital status, employment, and education level. Additionally, Spearman’s rho correlation coefficient was calculated to explore the association between patient age and the intensity of the reported symptoms.

The normality of the data distribution was assessed using the Kolmogorov–Smirnov test. Given the non-normal distribution of the ESAS-R scores, non-parametric methods were utilized for all inferential analyses. The Wilcoxon signed-rank test was used to compare symptom scores between Measurement A and Measurement B. This analysis was conducted on the matched longitudinal sample of 105 patients who completed both assessments. The Mann–Whitney U test was performed to compare median symptom values between two independent groups, such as gender or previous chemotherapy experience, while the Kruskal–Wallis H test was used for variables with more than two categories, including marital status, employment, and education level. Additionally, Spearman’s rho correlation coefficient was calculated to explore the association between patient age and the intensity of the reported symptoms.

Finally, binary logistic regression models were developed for both measurement periods to identify predictors of symptom intensity. The Enter method was used for variable selection, and model fit was evaluated using the Hosmer–Lemeshow test and Nagelkerke R^2^.

### 2.4. Ethics

This study was conducted in accordance with the Declaration of Helsinki and approved by the Assembly of International Hellenic University (7/17-3-21). Participation was entirely voluntary, and all participants were provided with a clear explanation regarding the purpose of the research, the confidentiality of their data, and their right to withdraw at any stage without affecting their medical care. Prior to data collection, written informed consent was obtained from each patient, ensuring that they understood the study’s requirements and the intended use of the information provided. To maintain anonymity, all personal identifiers were removed from the questionnaires, and the collected data were stored securely, accessible only to the research team for the purposes of statistical analysis.

## 3. Results

### 3.1. Demographic and Clinical Data of the Participants

A total of 189 participants completed the baseline assessment, whereas 105 completed the follow-up assessment. Longitudinal analyses were based on complete-case analysis, including only participants with complete data for both measurement points.

The study population was predominantly female, married, and urban dwelling, with breast cancer serving as the most frequent diagnosis at both Measurement A (59.3%) and Measurement B (64.8%). While most patients were not newly diagnosed, there was a significant increase in the proportion of participants with previous chemotherapy experience, which rose from 3.7% to 29.5% by the final measurement. Treatment complexity shifted notably over time, as the majority of patients moved from a two-drug chemotherapy regimen (66.1%) at the start to a single-drug regimen (42.9%) by the end of the study. Despite these changes in drug protocols, the method of administration remained highly consistent, with approximately 90% of treatments delivered via peripheral venous catheter in both phases. Additionally, hypertension was identified as the most common comorbidity, though the percentage of patients reporting no co-existing conditions increased slightly to 46.7% by the final measurement (Table 1).

### 3.2. Symptom Assessment

The Total Symptom Burden Score was calculated at the participant level by summing the nine core ESAS-R symptom ratings, excluding the “other problem” item. Descriptive statistics were calculated separately for Measurement A and Measurement B using the available observations at each measurement point. The mean Total Symptom Burden Score was 25.09 ± 8.32 at Measurement A and 24.19 ± 7.91 at Measurement B. The longitudinal comparison was performed using the 105 participants who completed both measurements. The Wilcoxon signed-rank test showed a marginally non-significant decrease in total symptom burden (*p* = 0.057).

Patient symptoms exhibited varying trends. The comparison between Measurement A and Measurement B was conducted on the matched longitudinal sample of 105 patients. Because symptom scores were not normally distributed, the Wilcoxon signed-rank test was used for paired comparisons. The main findings show that there was a statistically significant change (*p* < 0.05) only in pain, fatigue and well-being. Specifically, deterioration in physical condition was observed, as pain and fatigue showed an increase in mean intensity between the two measurements. At the same time, however, a significant deterioration was also noted in the sense of well-being (*p* < 0.05). In contrast, psychological symptoms (anxiety and depression) and other physical complaints (nausea and shortness of breath) did not show statistically significant differences (Table 2).

### 3.3. Univariate Analysis

Non-parametric tests were used to process the data, as the symptom data did not follow a normal distribution. Specifically, the Mann–Whitney U test was performed to compare the median values between two independent groups (e.g., gender, living alone, and previous chemotherapy) and the Kruskal–Wallis H test was performed to compare the median values between more than two groups (e.g., marital status, employment, and education). To investigate the relationship between age and symptom intensity, the Spearman’s rho correlation coefficient was used. For immunotherapy and combination of chemotherapy and targeted treatments, testing was performed only for the first measurement as in the last measurement all patients in the sample gave a negative response.

In Measurement A, the total score was primarily linked to treatment-related factors, specifically the chemotherapy regimen (*p* = 0.030) and the administration route (*p* = 0.030). By Measurement B, demographic and clinical factors became dominant, with education level (*p* = 0.010), previous chemotherapy history (*p* = 0.040), and combined immunotherapy (*p* = 0.040) showing significant associations with the total symptom score (Table 3).

The statistically significant association observed for chemotherapy combined with immunotherapy at Measurement B should be interpreted with caution because only two participants were classified in this category at follow-up, resulting in a very small cell count and an unstable estimate.

The correlation analysis (Table 4) revealed a consistent negative relationship between age and several physical symptoms. Specifically, age showed a consistent negative correlation with pain (*p* < 0.001) in the second measurement and nausea across both measurements (*p* = 0.004 and *p* = 0.017). By the final measurement (B), age also showed a significant negative correlation with shortness of breath (*p* = 0.048). At Measurement B, age was negatively correlated with the total symptom score (rho = −0.197, *p* = 0.044), whereas this association was not statistically significant at Measurement A (rho = −0.110, *p* = 0.134). Psychological symptoms (anxiety and depression) and well-being did not show any statistically significant correlation with age in either phase of the study.

### 3.4. Logistic Regression Analysis

To determine the factors affecting symptom intensity, a multivariate binary logistic regression was performed (Table 5). For the purposes of binary logistic regression, the total ESAS-R score was dichotomized into mild symptom burden (<40) and moderate-to-high symptom burden (≥40). This threshold was used as a study-specific operational definition corresponding approximately to an average score of four or higher across the nine core ESAS-R symptoms. It should not be interpreted as a universally validated cutoff for total ESAS-R symptom burden.

Optimal prediction models were developed for each measurement phase. Model goodness of fit was verified using the Hosmer–Lemeshow test, while interpretative ability was assessed via Cox & Snell R^2^ and Nagelkerke R^2^ indices. The “Enter” method was utilized for model development. Variables were selected for inclusion in the multivariable logistic regression models based on both statistical and clinical considerations. Variables showing an association with symptom burden at the univariate level (*p* < 0.05) were entered into the multivariable models. In addition, gender and living arrangement were retained as clinically relevant covariates because both may influence symptom perception, psychosocial support, and adjustment to cancer treatment and were therefore considered potential confounding factors.

For Measurement A, the model demonstrated a good fit (Hosmer–Lemeshow *p* = 0.288, Cox & Snell R^2^ = 0.071 and Nagelkerke R^2^ = 0.104). Among the variables examined, employment status (housework) was the only statistically significant predictor (*p* = 0.010). Specifically, patients in the housework category were significantly less likely to experience increased symptom severity (Exp(B) = 0.101, 95% CI: 0.018–0.578).

For Measurement B, the model also showed a good fit (Hosmer–Lemeshow *p* = 0.509, Cox & Snell R^2^ = 0.204 and Nagelkerke R^2^ = 0.318), explaining 20.4% to 31.8% of the variability in symptom severity. Baseline symptom burden category at Measurement A emerged as a significant predictor of symptom intensity at Measurement B (*p* = 0.028). Patients with mild symptom burden at Measurement A were less likely to report moderate-to-high symptom burden at Measurement B (Exp(B) = 0.11, 95% CI: 0.02–0.79). Male gender showed a trend toward significance but did not reach the *p* < 0.05 threshold (*p* = 0.058, Exp(B) = 0.10, 95% CI: 0.01–1.09). Other variables, including living situation and cancer type, did not show statistically significant effects in the final model.

## 4. Discussion

This prospective study underscores the complex and evolving nature of symptom burden in cancer patients under active treatment. The findings revealed a significant deterioration in pain, fatigue, and perceived well-being by the final chemotherapy cycle, although the overall symptom burden remained relatively stable. Furthermore, the findings highlight a critical transition in symptom predictors, shifting from treatment-related factors in early cycles to demographic influences, specifically younger age and lower education levels, as patients approach the end of their therapeutic journey.

Over the last three decades, the ESAS-R has become one of the most frequently utilized symptom scales for individuals with cancer [14]. Its psychometric characteristics in a diverse cancer population are strongly validated, demonstrating reliability and validity across various contexts [14,15]. It allows personalized symptom management in hospital and community settings, and healthcare professionals can reliably monitor patient symptoms in real time [16]. Therefore, the ESAS-R was appropriately utilized in the present study, as it represents a reliable and valid tool that effectively supports the assessment of patients undergoing active treatment and serves the aims of our research.

Although the total symptom burden changed only modestly, decreasing from 25.09 to 24.19 (*p* = 0.057), significant increases in pain and fatigue were observed. These increases were offset by numerical decreases in several other symptoms, including drowsiness, nausea, depression, and anxiety, resulting in a relatively stable overall total score. This suggests that changes in individual symptoms did not translate into a statistically significant worsening of the overall symptom score [17,18].

The main findings reveal a statistically significant increase in pain and fatigue between the two measurements, a trend consistent with previous research in Greece by Lavdaniti et al. (2018, 2021), which identified these symptoms as primary factors of burden in oncology patients [19,20]. Fatigue is also widely recognized as one of the most common symptoms among patients receiving active cancer treatment, particularly among breast cancer patients [21,22,23,24]. In the present study, the significant worsening in perceived well-being further supports the clinical relevance of the observed symptom changes [25,26].

In contrast, psychological symptoms (anxiety and depression) and other physical complaints (nausea and shortness of breath) did not show statistically significant differences. This is further supported by Lambert et al. (2026), whose recent validation study confirms the ongoing diagnostic accuracy of the ESAS-R as a screening tool for anxiety and depression in cancer populations [18]. This reinforces the validity of the stable psychological profiles observed in our sample from the third to the final chemotherapy cycle [18].

It is important to highlight that while the total score did not increase significantly, the pattern of individual symptoms changed during treatment. Pain and fatigue increased significantly, while several other symptoms showed decreases. Thus, the non-significant change in total score appears to reflect offsetting changes across individual symptoms rather than an absence of clinically relevant symptom changes.

While several symptoms reached statistical significance, it is vital to interpret these through the lens of the Minimal Clinically Important Difference (MCID). According to Hui et al. (2015), a 1-point shift on the ESAS scale signifies a change that is personally meaningful to the patient [27]. In our study, the increase in pain intensity met this threshold (mean increase of 1.0), confirming that the physical toll of cumulative chemotherapy cycles results in a clinically significant burden that requires proactive management.

Notably, a relative stability of shortness of breath was observed, which maintained an unchanged mean throughout the treatment trajectory. This finding aligns with the findings of Shin et al. (2023) [28]. Their research identified that a majority of oncology outpatients undergoing chemotherapy maintain a low-stable breathlessness profile across treatment cycles [28]. However, this stability in our sample may also be partially attributed to the composition of the population, as only 14 patients (7.4% of the initial sample) were diagnosed with lung cancer. In contrast, research specifically targeting advanced lung cancer in Greece [20] identified respiratory distress as a primary and highly prevalent driver of symptom burden and decreased quality of life.

While shortness of breath did not emerge as a dominant concern for our general sample, the negative correlation between age and shortness of breath noted in our final measurement is in line with the observations of Shin et al. (2023) that younger age is often associated with more severe symptom burden [28]. This suggests that younger participants reported higher intensity, yet the low representation of primary lung malignancies likely prevented this symptom from reaching the levels of severity observed in specialized respiratory oncology studies. Consequently, these findings emphasize that while shortness of breath may appear stable at a population level, it remains a critical marker for targeted monitoring and vigilant nursing care in younger patients and high-risk respiratory subgroups.

All of the significant correlations of the demographic and clinical variables with age were negative. This shows that younger patients in this study reported higher symptom burdens for pain, nausea, and overall symptoms compared to older patients. The relationship between age and pain actually grew stronger over time, moving from a weak correlation to a moderate one by the final measurement. On the other hand, psychological symptoms (anxiety and depression) and well-being did not show any statistically significant correlation with age in either phase of the study. These findings are consistent with previous studies [29,30,31,32] that noted that younger patients often experience higher levels of physical symptoms compared to elderly patients, who may possess lower physiological reactivity.

However, the specific lack of correlation between age and psychological symptoms in our findings match the observations of Morse et al. (2026) [33], who argued that psychological distress often does not follow the same age-dependent trajectories as physical toxicity. Their research suggests that while younger patients may report more severe physical symptoms, such as the increased pain and nausea clearly reflected in our findings, the psychological impact of cancer remains a universal burden that transcends age groups, potentially due to varying emotional regulation strategies and coping mechanisms developed across the lifespan [33]. Thus, mental health screening, with the range of mental health assessment instruments that exist, should be mandatory for all patients, regardless of age [34].

Moreover, the finding that younger patients reported higher physical symptom intensity for pain and nausea may be attributed to a higher degree of life disruption [35]. Younger individuals often balance active professional lives and caregiver roles for young children, which can lower their threshold for symptom tolerance compared to older patients who may have more established or different life priorities [36]. This suggests that for younger patients, symptom burden is not merely a biological response to chemotherapy, but a multifactorial experience influenced by social and developmental roles [33].

Educational level was significantly associated with total symptom burden at the final assessment in the univariate analysis. However, this association was not confirmed as an independent predictor in the multivariable logistic regression model. Therefore, the observed relationship between education and symptom burden should be interpreted as an unadjusted association rather than an independent predictive effect. This is similar to the studies of Gavala et al. (2026) [24] and Fradelos et al. (2026) [37], who found that socioeconomic factors and health literacy significantly impact treatment satisfaction and perceived symptom burden in Greek day-care units. The shift towards educational level as a predictor by the final cycle highlights the role of health literacy. Patients with limited health literacy tend to have less favorable outcomes [38]. While higher education often provides the vocabulary for early symptom identification and better access to supportive strategies, patients with lower education may face a cumulative burden due to challenges in navigating self-management protocols. This suggests that as treatment progresses, a patient’s internal resources and ability to decode medical information become more influential than the clinical regimen itself [39]. To address health literacy problems, oncology nurses should use diverse educational materials that rely on visual aids and simplified terminology [40].

At the early treatment phase (Measurement A) employment/housework status was the main protecting factor for increased symptom intensity as found in the regression analysis. So, at first, the role of employment and housework status suggests the importance of daily life continuation, engaging in familiar, productive roles that provide a sense of normalcy. Engaging in domestic responsibilities likely provides patients with a vital sense of normalcy or a sense that patients maintain their pre-diagnosis identity [41].

However, by the final cycle, the chemotherapy cycle itself, representing cumulative toxicity, becomes the dominant predictor. As cancer treatment moves on to completion, nurses have to deal with managing the toxicities and symptoms with acute medical and nursing interventions as well as holistic, person-centered care [42]. As patients reach their treatment’s end, the physiological burden of the drugs, as evidenced by the statistically significant increase in pain and fatigue, overrides the protective influence of socio-environmental factors (like employment). In this stage, a holistic approach is critical, as patients frequently report that their psychological and other supportive care needs are often unmet in the post-treatment phase. Oncology nurses must work on this, from strict management of drug toxicity in the first cycles, to psychosocial support and education in the final cycles [43].

In summary, our results validate and extend the existing Greek literature by shifting the focus from cross-sectional “snapshots” to a prospective understanding of how these determinants evolve over the course of treatment. The ESAS-R is useful for rapid symptom screening in busy clinical environments [18], like the ones in Greece. However, in Greece, the integration of Patient-Reported Outcome Measures (PROMs) into routine clinical care is currently in a transitional phase, moving from purely academic research toward organized, system-wide implementations [44]. Staffing shortages and high patient volume in tertiary Greek hospitals [45] make the routine collection and interpretation of PROMs challenging. Nevertheless, although significant challenges remain, the growing body of evidence from Greek studies suggests that meaningful efforts are underway toward the systematic integration of PROMs into oncology nursing practice.

The generalizability of these findings should be interpreted with caution. Participants were recruited from a single tertiary oncology hospital in Greece using convenience sampling, and breast cancer patients comprised the largest diagnostic group. Consequently, the findings may not be directly applicable to patients treated in other healthcare settings, different countries, or populations with different cancer distributions. Nevertheless, the prospective design and inclusion of patients with various malignancies enhance the relevance of the findings for outpatient chemotherapy settings with similar organizational characteristics.

### 4.1. Strengths and Limitations

This study presents several strengths, especially its prospective, longitudinal design, which allowed for the tracking of symptom evolution in the same patient cohort from the early stages (third cycle) to the completion of chemotherapy. The use of the ESAS-R, a globally validated and highly reliable tool in oncology and palliative care, ensured the systematic and consistent recording of a wide array of physical and psychological distress markers.

However, certain limitations must be acknowledged. The study was conducted at a single tertiary cancer hospital, which may limit the generalizability of the findings. In addition, the relatively high attrition rate (44.4%) represents an important limitation. Although the non-responder analysis did not identify significant differences between participants who completed follow-up and those who did not in terms of age, gender, or baseline total symptom burden, other clinically relevant characteristics, including cancer type, treatment regimen, and educational level, were not included in this comparison. Therefore, residual selection and attrition bias cannot be completely excluded.

Although non-responder analysis showed no statistically significant differences between completers and non-completers in age, gender, or baseline total symptom burden, residual attrition bias cannot be fully excluded. In addition, symptom assessment was based on self-report measures, which may introduce subjective reporting bias; however, patient-reported outcomes remain essential for capturing the patients’ direct symptom experience.

The explanatory power of the logistic regression models was modest, particularly at Measurement A (Nagelkerke R^2^ = 0.104), while the model at Measurement B explained a larger but still limited proportion of the variability (Nagelkerke R^2^ = 0.318). These findings indicate that important determinants of symptom burden were not captured by the variables included in the models, and therefore the predictive findings should be interpreted cautiously.

Additional sources of bias should also be considered. The use of convenience sampling from a single tertiary oncology hospital may have introduced selection bias and may limit the representativeness of the study population. Information bias is also possible because symptom assessment relied on patient self-report using the ESAS-R questionnaire. Although a non-responder analysis suggested that attrition did not introduce substantial systematic bias, residual attrition bias cannot be completely excluded. Furthermore, complete-case analysis was performed without imputation, and the frequency of missing data for individual variables could not be retrospectively quantified, limiting the assessment of the potential impact of missing data on the findings. Finally, residual confounding from unmeasured clinical variables cannot be excluded.

### 4.2. Implications for Nursing Practice

The study’s findings highlight a critical need for age-specific nursing interventions, as younger patients consistently reported higher intensity in symptoms such as pain and nausea across both measurement periods. In the Greek oncology setting, where patient loads are often high, nurses should prioritize younger cohorts for more frequent symptom screening and aggressive personalized pain management protocols. This negative correlation between age and physical distress suggests that younger individuals may have different physiological responses or higher expectations for quality of life during treatment, requiring nurses to act as proactive advocates for their supportive care needs.

Also, the importance of tailored patient education is evident. Nurses in Greek hospitals should move beyond standardized information leaflets and adopt more accessible, simplified communication strategies for patients with lower educational backgrounds to ensure they can identify and report symptoms effectively. Since the Total Symptom Score showed a transition from being influenced by clinical treatment factors (like drug regimens) to being influenced by demographic factors (like education) over time, nursing care must evolve from a technical focus in early cycles to a more holistic, person-centered approach in the later stages.

The reported symptoms affect patients’ well-being. Thus, nursing practice should address the late-stage deterioration in perceived well-being by reinforcing psychological support and preparing patients for the transition toward survivorship care. Given that the majority of treatments in this setting are administered via peripheral catheters, nurses must also maintain high standards of vascular access care to prevent additional physical distress, while simultaneously involving the patient’s family, a cornerstone of Greek culture, in the management of psychological symptoms like anxiety and depression.

### 4.3. Future Directions

Future research should prioritize longitudinal studies with larger, more diverse cohorts to validate the shifting influence of demographic factors, such as education and age, on symptom trajectories across different cancer types and treatment modalities. Investigating the specific barriers faced by younger patients and those with lower educational backgrounds could facilitate the development of targeted, culturally sensitive educational programs within the Greek healthcare system. Future studies could also investigate the integration of electronic ESAS-R administration and real-time symptom monitoring into routine oncology care.

From an educational perspective, nursing curricula and hospital training programs should emphasize the systematic use of validated assessment tools like the ESAS-R, moving beyond clinical drug-focused care toward a more holistic, person-centered approach that addresses both somatic toxicity and psychological adaptation. Additionally, exploring the integration of digital health platforms for real-time symptom monitoring could enhance early intervention and improve the long-term quality of life for oncology patients transitioning into survivorship.

## 5. Conclusions

This longitudinal study demonstrates that pain, fatigue, and perceived well-being significantly deteriorated by the final chemotherapy cycle, although the overall symptom burden remained relatively stable.

The findings suggest that younger age and lower educational level may be associated with higher symptom burden, particularly in the later stages of chemotherapy. This highlights the need for personalized, person-centered oncology nursing interventions tailored to patients’ age, educational needs, and treatment phase. These findings underscore the importance of utilizing validated tools like the ESAS-R in Greek clinical settings to facilitate targeted interventions that address the evolving somatic and emotional needs of patients throughout their cancer journey.

## Figures and Tables

**Table 1:** Demographic and clinical data of the participants. Measurement A included all baseline participants (*n* = 189), whereas Measurement B included the longitudinal follow-up sample (*n* = 105).

Variable	Category	Measurement A (*n*, %)		Measurement B (*n*, %)	
Gender	Male	43	22.8%	21	20.0%
	Female	146	77.2%	84	80.0%
Marital status	Unmarried	19	10.1%	10	9.5%
	Married	135	71.4%	73	69.5%
	Divorced	15	7.9%	8	7.6%
	Widowed	18	9.5%	13	12.4%
	Cohabitation	2	1.1%	1	1.0%
Living alone	Yes	56	29.6%	20	19.0%
	No	133	70.4%	85	81.0%
Employment status	Unemployed	19	10.1%	9	8.6%
	Private Employee	30	15.9%	15	14.3%
	Public Employee	15	7.9%	11	10.5%
	Household	33	17.5%	25	23.8%
	Retiree	83	43.9%	42	40.0%
	Other	9	4.8%	3	2.9%
Insurance status	Public	172	91.0%	96	91.4%
	Private	4	2.1%	1	1.0%
	Public And Private	2	1.1%	1	1.0%
	Uninsured	11	5.8%	7	6.7%
Education	Illiterate	1	0.5%	1	1.0%
	Primary School	54	28.6%	26	24.8%
	Middle School	33	17.5%	14	13.3%
	High School	70	37.0%	40	38.1%
	Secondary Education	10	5.3%	7	6.7%
	Technical Institute	4	2.1%	5	4.8%
	University	16	8.5%	11	10.5%
	Postgraduate	1	0.5%	1	1.0%
Place of residence	Urban	136	72.0%	71	67.6%
	Semi-Urban	42	22.2%	22	21.0%
	Rural	11	5.8%	12	11.4%
Cancer type	Breast Cancer	112	59.3%	68	64.8%
	Lung Cancer	14	7.4%	6	5.7%
	Colorectal Cancer	17	9.0%	8	7.6%
	Pancreatic Cancer	3	1.6%	1	1.0%
	Hepatocellular Carcinoma	1	0.5%	1	1.0%
	Prostate Cancer	7	3.7%	8	7.6%
	Kidney Cancer	1	0.5%	-	-
	Endometrial Cancer	7	3.7%	2	1.9%
	Sarcomas	2	1.1%	1	1.0%
	Other	18	9.5%	5	4.8%
	Melanoma	1	0.5%	-	-
	Ovarian Cancer	6	3.2%	5	4.8%
Comorbidities	None	81	42.9%	49	46.7%
	Hypertension	45	23.8%	18	17.1%
	Diabetes Mellitus	13	6.9%	9	8.6%
	Heart Failure	3	1.6%	-	-
	Respiratory Disease	2	1.1%	1	1.0%
	Autoimmune Disease	1	0.5%	1	1.0%
	Other	44	23.3%	27	25.7%
Administration route of treatment	Peripheral Catheter	173	91.5%	94	89.5%
	Central Venous Line	4	2.1%	3	2.9%
	Port-a-Cath	8	4.2%	8	7.6%
	Portable Pump 48 h	4	2.1%	-	-
Chemotherapy regimen	1 Drug	37	19.6%	45	42.9%
	2 Drugs	125	66.1%	41	39.0%
	3 Drugs	23	12.2%	15	14.3%
	4 Drugs	4	2.1%	4	3.8%
Newly diagnosed patient	Yes	37	19.6%	16	15.2%
	No	152	80.4%	89	84.8%
Previous chemotherapy	Yes	7	3.7%	31	29.5%
	No	182	96.3%	74	70.5%
Chemotherapy & targeted therapy	Yes	4	2.1%	-	-
	No	185	97.9%	105	100.0%
Chemotherapy & immunotherapy	Yes	1	0.5%	2	1.9%
	No	188	99.5%	103	98.1%
Immunotherapy	Yes	2	1.1%	-	-
	No	187	98.9%	105	100.0%

Note: Measurement A represents baseline assessment after the third chemotherapy cycle, whereas Measurement B represents the follow-up assessment after the final chemotherapy cycle. Demographic characteristics such as sex, marital status, education, and place of residence are presented as participant characteristics; treatment-related variables may have changed between measurements.

**Table 2:** Descriptive measures of ESAS-R items.

Symptom	Measurement A (Mean ± SD)	Measurement B (Mean ± SD)	*p*-Value
Pain	2.19 ± 2.75	3.19 ± 2.91	0.002
Tiredness (lack of energy)	4.1 ± 2.7	4.6 ± 2.7	0.001
Drowsiness (feeling sleepy)	2.6 ± 2.8	2.2 ± 2.6	0.970
Nausea	2.2 ± 2.8	1.6 ± 2.4	0.117
Lack of appetite	2.6 ± 3.0	2.4 ± 2.7	0.051
Shortness of breath	1.1 ± 2.2	1.1 ± 2.1	0.092
Depression (feeling sad)	3.1 ± 2.9	2.7 ± 2.5	0.323
Anxiety (feeling nervous)	3.4 ± 3.0	3.0 ± 2.6	0.482
Well-being (overall feeling)	3.7 ± 2.6	4.3 ± 2.5	0.006
Other problem	2.6 ± 3.0	2.1 ± 2.8	0.545
Total Symptom Burden Score	25.09 ± 8.32	24.19 ± 7.91	0.057

Note: Individual ESAS-R item means were calculated using available responses for each item; no missing values were imputed. The Total Symptom Burden Score was calculated at the participant level by summing the nine core ESAS-R items. Therefore, the number of valid observations contributing to individual item means may differ from that contributing to the total score.

**Table 3:** Statistical analyses (*p*-values) per variable.

Variable	Statistical Test	Total Score (p) Measurement A	Total Score (p) Measurement B	Significant Individual Symptoms (*p*-Value)
Gender	Mann–Whitney U	0.950	0.700	Nausea: A: 0.03/B: 0.24 Shortness of Breath: A: 0.01/B: 0.49
Marital Status	Kruskal–Wallis H	0.160	0.990	Appetite Loss: A: 0.09/B: 0.45
Living Alone	Mann–Whitney U	0.180	0.220	Depression: A: 0.03/B: 0.49 Well-being: A: 0.08/B: 0.17
Employment	Kruskal–Wallis H	0.160	0.260	Nausea: A: 0.01/B: 0.48 Pain: A: 0.06/B: 0.05
Insurance Status	Kruskal–Wallis H	0.390	0.440	Appetite Loss: A: 0.10/B: 0.23 Shortness of Breath: A: 0.06/B: 0.83
Education	Kruskal–Wallis H	0.210	0.010 *	Fatigue: A: 0.09/B: 0.04 Pain: A: 0.38/B: 0.06
Residence	Kruskal–Wallis H	0.420	0.740	Drowsiness: A: 0.93/B: 0.04
Cancer Type	Kruskal–Wallis H	0.650	0.090	Shortness of Breath: A: 0.01/B: 0.18 Pain: A: 0.64/B: 0.02
Comorbidities	Kruskal–Wallis H	0.130	0.230	Drowsiness: A: 0.09/B: 0.43 Shortness of Breath: A: 0.88/B: 0.10
Administration Route	Kruskal–Wallis H	0.030 *	0.110	Drowsiness: A: 0.02/B: 0.61 Appetite Loss: A: 0.00/B: 0.25
Chemotherapy Regimen	Kruskal–Wallis H	0.030 *	0.270	Pain: A: 0.02/B: 0.17 Well-being: A: 0.01/B: 0.39
Chemotherapy Cycle	Mann–Whitney U	0.120	0.780	Nausea: A: 0.08/B: 0.45
Chemotherapy & Immunotherapy	Mann–Whitney U	0.760	0.040 *	Drowsiness: A: 0.10/B: 0.13 Well-being: A: 0.60/B: 0.13
Immunotherapy	Mann–Whitney U	0.970	**-**	Well-being: A: 0.05/B: -
Previous Chemotherapy	Mann–Whitney U	0.570	0.040 *	Pain: A: 0.17/B: 0.03 Fatigue: A: 0.98/B: 0.05
Newly Diagnosed Patient	Mann–Whitney U	0.090	0.070	Appetite Loss: A: 0.00/B: 0.05 Well-being: A: 0.89/B: 0.00
Chemotherapy & Targeted Therapy	Mann–Whitney U	0.900	**-**	Depression: A: 0.34/B: -

Notes: (*): statistical significance at the *p* < 0.05 level; (-): Indicates the analysis was only available for one measurement period.

**Table 4:** Spearman’s rho correlations for age (A: *n* = 189, B: *n* = 105).

Symptom	Measurement A (rho)	*p*-Value (A)	Measurement B (rho)	*p*-Value (B)
Pain	−0.167 *	0.022	−0.309 **	<0.001
Tiredness	−0.090	0.231	−0.180	0.064
Drowsiness	−0.080	0.291	−0.110	0.273
Nausea	−0.211 **	0.004	−0.233 *	0.017
Lack of Appetite	0.050	0.501	0.010	0.942
Shortness of Breath	0.010	0.892	−0.194 *	0.048
Depression	0.001	0.963	−0.150	0.132
Anxiety	−0.020	0.831	0.040	0.721
Well-being	0.040	0.602	0.060	0.564
Other Problem	−0.130	0.081	0.010	0.893
Total Symptom Score	−0.110	0.134	−0.197 *	0.044

Notes: rho: Spearman’s correlation coefficient; (*): correlation is significant at the 0.05 level (2-tailed); and (**): correlation is significant at the 0.01 level (2-tailed).

**Table 5:** Logistic regression models for symptom intensity (Measurements A and B).

Variable	B	S.E.	Wald	df	Sig. (p)	Exp(B)	95% CI for Exp(B)
Measurement A							
Gender (male)	0.202	0.564	0.128	1	0.720	1.223	0.405–3.694
Living alone (yes)	0.177	0.384	0.213	1	0.644	1.194	0.563–2.535
Employment (housework)	−2.292	0.890	6.641	1	0.010 *	0.101	0.018–0.578
Residence (semi-urban)	0.563	0.907	0.385	1	0.535	1.755	0.297–10.381
Constant	1.100	1.561	0.496	1	0.481	3.003	—
Measurement B							
Gender (male)	−2.26	1.20	3.58	1	0.058	0.100	0.010–1.090
Living alone (yes)	0.62	0.71	0.76	1	0.383	1.850	0.460–7.370
Cancer type (breast cancer)	0.82	0.62	1.74	1	0.187	2.270	0.670–7.690
Baseline symptom burden category	−2.18	0.99	4.84	1	0.028 *	0.110	0.020–0.790
Constant	1.86	2.67	0.48	1	0.486	6.4000	—

Notes: (*) Statistical significance at the *p* < 0.05 level. Dependent variable: Total Symptom Burden Score categorized as 0 = mild symptom burden (<40) and 1 = moderate-to-high symptom burden (≥40). Abbreviations: B: regression coefficient; S.E.: standard error; Wald: Wald chi-square statistic; df: degrees of freedom; Sig.: *p*-value; and Exp(B): odds ratio. Model fit (Measurement A): Hosmer and Lemeshow test: χ^2^ = 9.692, *p* = 0.288; Cox & Snell R^2^ = 0.071; and Nagelkerke R^2^ = 0.104. Model fit (Measurement B): Hosmer and Lemeshow test: χ^2^ = 7.265, *p* = 0.509; Cox & Snell R^2^ = 0.204; and Nagelkerke R^2^ = 0.318. For Measurement B, baseline symptom burden category refers to the total ESAS-R symptom burden classification at Measurement A.

## Data Availability

The data supporting the findings of this study are available upon request from the corresponding author due to privacy or ethical restrictions.

## Public Involvement Statement

No patients or members of the public were involved in any aspect of this research.

## Guidelines and Standards Statement

No specific reporting guideline was used in the preparation of this manuscript.
